# Effects of foreign immigrants on malaria situation in cleared up and potential foci in one of the highest malaria burden district of southern Iran

**DOI:** 10.1186/1475-2875-11-S1-P81

**Published:** 2012-10-15

**Authors:** Ahmad Raiesi, Jalil Nejati, Alireza Ansari-moghaddam, Mohammad Sakeni, Leila Faraji, Bita Paktinat, Fatemeh Nikpour, Farideh Kamali, Faranak Raiesi

**Affiliations:** 1 School of Public Health, Tehran University of Medical Sciences and national program manager for malaria control, MOH&ME,Tehran,IR Iran; 2 Disease Control & Prevention, Province Health Center, Zahedan, Iran; 3 Health Promotion Research Center, Zahedan University of Medical Sciences, Iran; 4 National Program for Malaria Control, Center of Disease Control & Prevention, Ministry of Health and Medical Education, Tehran, Iran; 5 Shahid Beheshti University, Ministry of High Education, Tehran, Iran

## Background

Objective: The one of the main objectives of malaria elimination program is protection and expansion of cleared up foci, towards and its final goal which is zero is to cut off the indigenous malaria cases. This study is aimed to assess the effect of foreign immigrants on malaria incidence in some clear up and potential foci in Konarak District, south east of Iran.

## Material and methods

In this descriptive-analytic study, the numbers of malaria patients in clear up and potential foci were analyzed in Jahliyan region, located on the route of Pakistani and Afghan migration immigrants, during the 2005 to 2009. Data were described using frequency tables and analyzed by paired T-test. Also some of the development indicators were investigated in order to make sure that they did not change during the years of the study period.

## Results

The Annual Parasite Incidence (API) increased from a range of “30 to 142.9” after presence of immigrants in 2007, while it was “0 to 49” three years before their presence. The paired T-test showed a significant difference between the number of malaria cases in the villages from 2006 to 2008 and also 2007 to 2008. Development indicators didn’t have dramatic change during the five years, 2005-09 years.

## Conclusions

According to this research, the major cause of increasing malaria in the villages was the presence of foreign immigrants that led to increasing API index in 2008; so, cross border movement foreign immigration is a critical issue point to be considered in the malaria elimination program especially in the cleared up foci.

